# An Exploratory Study of PN HPT for Treating Postsurgical Atrophic and Depressed Scars

**DOI:** 10.1111/jocd.16764

**Published:** 2025-01-15

**Authors:** Antonino Araco, Francesco Araco, Mauro Raichi

**Affiliations:** ^1^ Cosmetic and Plastic Surgeon, Aesthetic Medicine Department Tor Vergata University Rome Italy; ^2^ Cosmetic and Plastic Surgeon San Giovanni Evangelista Hospital Tivoli Italy; ^3^ Clinical Pharmacology Consultant in Aesthetic Medicine Milan Italy

**Keywords:** Antera 3D CS, Global Aesthetic Improvement Scale, modified Vancouver Scar Score, PN HPT, Polynucleotides High Purification Technology, postsurgical mammary scars

## Abstract

**Background:**

Postsurgical atrophic scars tend to respond poorly to treatments, especially non‐energy‐based ones. Hydrophilic PN HPT (Polynucleotides High Purification Technology) injected intradermally is a non‐energy‐based option with an immediate volume‐enhancing effect that indirectly improves the fibroblast synthesis of collagen and extracellular matrix. The PN HPT ingredient has the further benefit of a dermal “priming” effect that enhances the efficacy of other scar treatments.

**Objectives:**

Verify retrospectively, with advanced techniques, the efficacy of PN HPT monotherapy as postsurgical scar treatment.

**Methods:**

Retrospective data collection in 18‐ to 65‐year‐old women with moderate‐to‐severe atrophic scars after mammary surgery undergoing a five‐session intradermal treatment course with 0.75% PN HPT gel formulation in single‐use syringes starting 6 months after surgery. Primary retrospective efficacy parameter: changes in scar morphology and symptom severity after three and 6 months (modified Vancouver Scar Scale, mVSS). Secondary efficacy parameters: roughness score 6 months after baseline (Antera 3D CS tridimensional skin analysis system) and Global Aesthetic Improvement Scale (GAIS, Investigator and Patient subscales) after three and 6 months.

**Results:**

Total mean mVSS highly significantly improved from 11.2 ± 1.92 at baseline to 7.0 ± 1.68 and 6.9 ± 1.55 after three and 6 months, respectively; the mean Antera 3D CS roughness score improved from 13.5 ± 4.14 to 10.0 ± 3.49 after 6 months. After three and 6 months, the GAIS subscores for investigators and cohort subjects were identical (3.0 ± 0.81 and 3.0 ± 0.72, respectively). The photographic documentation supported the previous results.

**Conclusions:**

In monotherapy, the intradermal PN HPT ingredient seems to quickly and safely relieve the burden of postsurgical atrophic scars. However, the lack of a formal parallel control group is a severe limitation. The objective quantitative measurements confirmed the long‐lasting benefits.

## 1 Background

A few days after surgery, the initially glistening surface of the surgical wound due to the hemostatic fibrin plug shifts to an eschar of dead tissue. Microscopically, neutrophils enter the fibrin‐rich surgical wound area to dispose of bacterial contaminants and tissue debris [1, 2]. About half a week after surgery, macrophages begin infiltrating the wound area, producing cytokines and growth factors that ignite the proliferation of granulation tissue, the meshwork of macrophages, fibroblasts, and endothelial cells that is the hallmark of the proliferative phase of scarring. Fibroblasts actively lay down type‐III collagen. At the same time, keratinocytes near the surgical wound edges migrate across the wound surface to close the skin defect [1, 2]. As a result, beginning after the third week and over the following year, the initially red and indurated cutaneous scar becomes softer and paler than the surrounding skin due to apoptosis of the granulation tissue cells and remodeling. Microscopically, parallel bundles of more solid type‐I collagen appear to replace the basket‐weave type‐III collagen fibers of the previous proliferative phase [1, 2].

The destruction of collagen following severe inflammation, as in surgical wounds or deep inflammatory acne, excavates the dermal depressions that are the hallmark of atrophic scars in their variable clinical presentations: narrow and sharply demarcated, V‐shaped ice pick scars extending deep into the dermis (estimated at 60%–70% of atrophic scars), wider U‐shaped scars with vertical edges extending 0.1–0.5 mm into the dermis (20%–30%), and wide‐surface rolling scars of irregularly undulating appearance with fibrous tethering of the dermis to the subcutis (15%–25%) [3, 4].

Subcision, or subcutaneous needle untethering of fibrous strands within the scar, (micro)dermabrasion, microneedling, dermal fillers, and chemical peels are examples of non‐energy‐based, superficial treatment options for depressed atrophic scars following surgical wounds [4]. Differently from energy‐based treatments like ablative and non‐ablative lasers, fractional radiofrequency, intense pulsed light, and plasma skin regeneration, superficial skin resurfacing benefits only shallow scars; all other presentations of atrophic scars are more resistant to superficial treatments [4].

Although diverse, all these superficial techniques stimulate the synthesis of new connective tissue; moreover, they avoid the risk of dyspigmentation, erythema, edema, scarring, and downtime after energy‐based procedures [4]. Unfortunately, with the partial exception of skin needling, post‐inflammatory hyperpigmentation may follow exposure to all non‐energy‐based techniques [5].

Natural‐origin polynucleotides (PN HPT, Polynucleotides High Purification Technology) injected intradermally are widely considered a safe alternative within the non‐energy‐based treatments of postsurgical scars [5]. PN HPT have also proved helpful as dermal priming agents before and concomitantly with other energy‐based and non‐energy‐based treatments to enhance the efficacy of those secondary treatments: a strategy labeled PN HPT Dermal Priming Paradigm [6].

PN HPT are highly fragmented polynucleotide residues between 50 and 2000 base pairs, free from biologically active contaminants and devoid of direct pharmacological activities and allergic potential [6, 7, 8]. The PN HPT ingredient hydrates the dermal microenvironment with a short‐term tissue‐filling effect. Peculiarly, over the longer term, the PN HPT ingredient of the intradermal PLINEST fast medical device physiologically activates dermal fibroblasts via the passive replenishment of the tissue pools of nitrogen bases and nucleotide precursors [6, 7, 8]. The two actions are synergic since hydration of the extracellular matrix is an essential prerequisite for fibroblast vitality; moreover, the fibroblast‐activating properties are the foundations of the priming strategy before or concomitantly with other treatments [6, 7]. The increased deposition of type‐I collagen, elastin fibers, and extracellular matrix is the hallmark of fibroblast activation [6, 7].

The retrospective exploratory study herein described aimed to confirm the benefits of PN HPT intradermal treatment on atrophic depressed scars following surgery in mammary areas after 6 months of follow‐up. A distinctive feature of the study was the quantitative, objective assessment to minimize any subjective bias by investigators.

## 2 Materials and Methods

### 2.1 Study Design and Patients

Retrospective open‐label data collection, with the preliminary signed consent of participating subjects before starting all procedures, in office‐treated non‐pregnant or breastfeeding women 18–65 years old with moderate‐to‐severe atrophic scars after mammary surgery (grades 3 and 4 of the Goodman and Baron semiquantitative scar grading scale; Table 1) [9].

**TABLE 1:** Descriptors of the Goodman and Baron semiquantitative scar grading scale [9].

Grade	Disease level	Clinical features
1	Macular disease	Erythematous, hyper‐ or hypopigmented flat marks visible to the patient or observer irrespective of distance
2	Mild disease	Mild atrophy or hypertrophic scars that may not be obvious at social distances of 50 cm or greater and may be covered adequately by makeup or the normal shadow of shaved beard hair in males, or normal body hair if extrafacial
3	Moderate disease	Moderate atrophic or hypertrophic scarring that is obvious at social distances of 50 cm or greater and is not covered easily by makeup or the normal shadow of shaved beard hair in males or body hair if extra facial, but is still able to be flattened by manual stretching of the skin (if atrophic)
4	Severe disease	Severe atrophic or hypertrophic scarring that is obvious at social distances greater than 50 cm and is not covered easily by makeup or the normal shadow of shaved beard hair in males or body hair if extra facial and cannot be flattened by manual stretching of the skin

Before the retrospective enrollment, cohort women should have never undergone postsurgical scar treatments and discontinued all dermatological treatments and procedures. Further exclusion criteria were the evidence of hypertrophic or keloid scars, severe solar elastosis or scarring, chronic drug or alcohol abuse, and hypersensitivity to the formulation ingredients. Beginning the restructuring PN HPT treatment was unrelated to the surgical procedure; a request by patients unsatisfied with the aesthetic situation or the clinician's advice led to the decision to begin the PN HPT treatment cycle. The period between surgery and PN HPT restructuring treatment was about 6 months.

Formal exclusion criteria did not include other dermatologic and systemic conditions. However, if present, such conditions deserved particular consideration in the retrospective analysis. Examples are the simultaneous intake of anticoagulant or antiplatelet medications; burns and discharging wounds still requiring a dressing 3 weeks after surgery or lacking visible signs of normal epithelialization; a history of autoimmune disease or other systemic diseases like diabetes mellitus, renal insufficiency, heart failure, hematologic, oncologic, psychiatric, and infectious diseases with related therapies.

### 2.2 Retrospective Study Procedures

Study formulation: PN HPT gel formulation (0.75% in phosphate buffer) in sterile, single‐use, 2‐mL pre‐filled glass syringes with two fine‐gauge 30G/13 mm needles (PLINEST fast, Mastelli, Sanremo, Italy). Office‐based treatment in the retrospective cohort of women: five intradermal administrations at baseline and after 2, 4, 6, and 8 weeks. Photographs of mature postsurgical scars, aged between 12 and 36 months, were taken in standardized conditions before the baseline injection, at the 3‐month assessment visit, and at the end of the 6‐month follow‐up period. A blinded evaluator, different from the operator, performed all assessments.

### 2.3 Primary Retrospective Efficacy Parameter

Retrospectively, the primary efficacy parameter was the changes in scar morphology assessed by a blind evaluator and the symptomatic impact three and 6 months after the baseline PN HPT injection, compared with baseline and scored according to a modified Vancouver Scar Scale (Table 2).

**TABLE 2:** Descriptors of the mVSS used in the study [10, 11].

Scar parameter		Score
Vascularity	Normal	0
	Pink	1
	Red	2
	Purple	3
Pigmentation	Normal	0
	Hypo‐pigmentation	1
	Mixed‐pigmentation	2
	Hyper‐pigmentation	3
Pliability (elasticity)	Normal	0
	Supple (flexible with minimal resistance)	1
	Yielding (giving way to pressure)	2
	Firm (inflexible, not easily moved, resistant to manual pressure)	3
	Banding (rope‐like tissue that blanches with extension of the scar)	4
	Contracture (permanent shortening of scar‐producing deformity or distortion)	5
Height	Flat	0
	Less than 2 mm	1
	2–5 mm	2
	More than 5 mm	3
Pain	None	0
	Occasional	1
	Requiring medication	2
Itchiness	None	0
	Occasional	1
	Requiring medication	2

The modified Vancouver Scar Scale (mVSS) of the study, an updated, validated version of the original Vancouver Scar Scale, is used to assess, semi‐quantitatively and subjectively, the severity of scars based on their appearance, texture, and related symptoms and to monitor their evolution over time. mVSS scores scar severity according to six morphological and clinical parameters—vascularity (scores 0 to 3), pigmentation (scores 0 to 3), flexibility/pliability (scores 0 to 5), height/thickness (scores 0 to 3), pain (scores 0 to 2), and itchiness (scores 0 to 2). The maximum total score is 18, with 0 indicating normal skin and 18 indicating extremely severe skin scarring and symptoms [10, 11].

### 2.4 Secondary Retrospective Efficacy Parameters

The first secondary retrospective endpoint was the variation of scar roughness, assessed by comparing objectively and quantitatively the baseline and sixth‐month photographs; the second one was the aesthetic improvement of the atrophic scars, subjectively scored by the investigators and treated subjects three and 6 months after the baseline injection versus baseline.

The tridimensional optical skin analysis system Antera 3D CS from Miravex Limited, Dublin, Ireland, a Trinity College spin‐off company, is based on a lightweight, handheld camera coupled with a digital‐analysis technology for photometric, color‐coded tridimensional skin reconstruction. Multiple bidimensional images are taken with different light sources at seven diode‐emitted wavelengths of different skin penetrations spanning most of the visible spectrum. The two‐dimensional images are then digitally processed to construct three‐dimensional images emphasizing, according to specific requirements, the pigment content, the texture/roughness, the density of capillaries, and other markers of scarring, making them objectively measurable and comparable [12, 13, 14].

The Global Aesthetic Improvement Scale (GAIS) is a validated five‐point scale widely used to rate global aesthetic improvements compared to baseline, assessed by the investigator and the treated subjects with specific and independent subscales.

The rating categories are “worse,” “no change,” “improved,” “much improved,” and “very much improved” (Table 3) [15]. GAIS assessments: three and 6 months after baseline.

**TABLE 3:** Descriptors of the Global Aesthetic Improvement Scale [15].

Rating	Description
5 Very much improved	Optimal cosmetic result in this patient
4 Much improved	Marked improvement in appearance from the initial condition, but not completely optimal for this patient. A touch‐up would slightly improve the result
3 Improved	Obvious improvement in appearance from the initial condition, but a touch‐up or retreatment is indicated
2 No change	The appearance is essentially the same as the original condition
1 Worse	The appearance is worse than the original condition

### 2.5 Safety Assessment

The incidence of adverse events throughout the study period, including severe events and infections, was retrospectively reported as a percent of the cohort patients based on spontaneous patient reporting and objective evaluations.

### 2.6 Sample Size and Statistical Analysis

Based on previous evidence from the “Preliminary Prospective and Randomised Study of Highly Purified Polynucleotide vs Placebo in Treatment of Moderate to Severe Acne Scars” study, the assumption of a 90% power of avoiding false‐negative type II errors (*ß* = 0.10) and the effect size for mVSS scores set at a conservative 0.2 (20% mean improvement in mVSS scores at the end of the treatment course), 30 subjects were estimated to be an adequate sample size [16]. Statistical program: GPower version 3.14 [17].

Descriptive statistics: means ± standard errors of the mean (SEM), medians, and 95% confidence intervals. Inferential statistics for the primary retrospective efficacy parameter are conservatively based on the non‐parametric general linear model for repeated measures in relatively few individuals (Kruskal–Wallis test for independent samples or non‐parametric one‐way ANOVA on ranks), aiming to assess how the PN HPT treatment influenced the within‐patient mVSS score evolution over time. After detecting a significant divergence from the null hypothesis (no treatment effect), pairwise post hoc Šidák multiple comparisons identified the exact point of divergence of the mVSS curve—three or 6 months after baseline [17].

Inferential statistics for the secondary retrospective efficacy parameters:

— After preliminary control for normal distribution (Kolmogorov–Smirnov test), paired‐sample Student's *t*‐test for variations in the within‐patient Antera 3D CS scar roughness score at 6 months vs. baseline.
— Non‐parametric Kruskal–Wallis test for independent samples for the Investigator and Patient GAIS subscore curves, followed by Šidák multiple comparisons in case of a significant divergence of the curves from the null hypothesis [18].

All statistical tests were two‐sided with a 5% significance level.

## 3 Results

### 3.1 Primary Retrospective Efficacy Parameter

Table 4 illustrates the demographics of the 30 adult women who satisfied the inclusion and exclusion criteria and were retrospectively enrolled. The final study cohort appeared non‐skewed, with all demographic parameters normally distributed.

**TABLE 4:** Characteristics of the cohort women; SEM = standard error of the mean.

Mean age (years ± SEM)^a^	39.9 ± 10.36
Range/median	24 to 61/37.0
Mean weight (kg)	66.9 ± 9.33
Range/median	47 to 93/68.0
Mean height (cm)^b^	169.8 ± 4.79
Range/median	160 to 185/170

^a^ Missing data for ten subjects.

^b^ Missing data for one subject.

**FIGURE 1:**
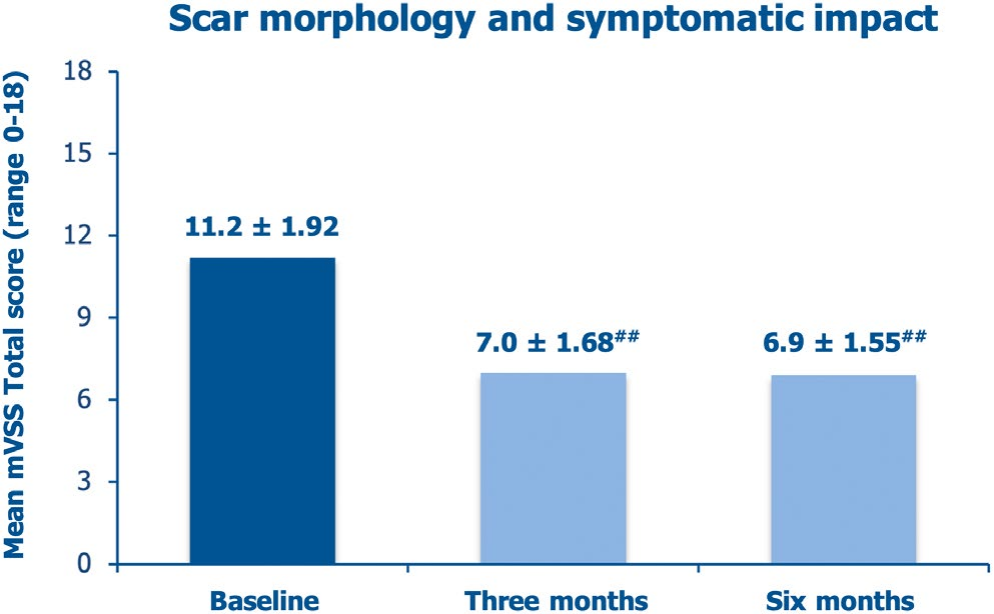
Mean mVSS total scores at baseline and after 3 and 6 months (^##^ *p* < 0.001 vs. baseline).

Regarding scar morphology and symptomatic impact, the total mean baseline mVSS was a severe 11.2 ± 1.92, decreasing with intradermal PN HPT treatment by 37.5% vs. baseline after 3 months and further marginally improving to −38.4% vs. baseline after 6 months (Figure 1). More analytically, Table 5 shows the highly significant improvements for almost all mean mVSS individual scores, including pain and itchiness. The retrospectively interviewed subjects reported that the improved pliability and elasticity, allowing the scars to move more easily with the surrounding normal skin, were the main reasons for the reduced, subjectively disturbing pain and discomfort. The investigators remarked that the improved pliability and elasticity of the scars translated into a reduced risk of complications such as nerve damage and contractures.

**TABLE 5:** mVSS subscores (mean ± SEM) at baseline and after 3 and 6 months.

Scar feature or symptom	Baseline	After 3 months	After 6 months
Vascularity	1.90 ± 0.76	1.80 ± 0.66	1.80 ± 0.66
Pigmentation	2.03 ± 0.56	1.83 ± 0.53*	1.77 ± 0.63*
Pliability/Elasticity	3.13 ± 0.43	1.83 ± 0.83^##^	1.90 ± 0.80^##^
Height	2.13 ± 0.73	1.37 ± 0.67^##^	1.37 ± 0.61^##^
Pain	1.00 ± 0.00	0.13 ± 0.35^##^	0.03 ± 0.18^##^
Itchiness	1.03 ± 0.18	0.03 ± 0.18^##^	0.00 ± 0.00^##^

Abbreviation: NS, not significant.

*p < 0.05 vs. baseline; ^##^ *p* < 0.001 vs. baseline.

**FIGURE 2:**
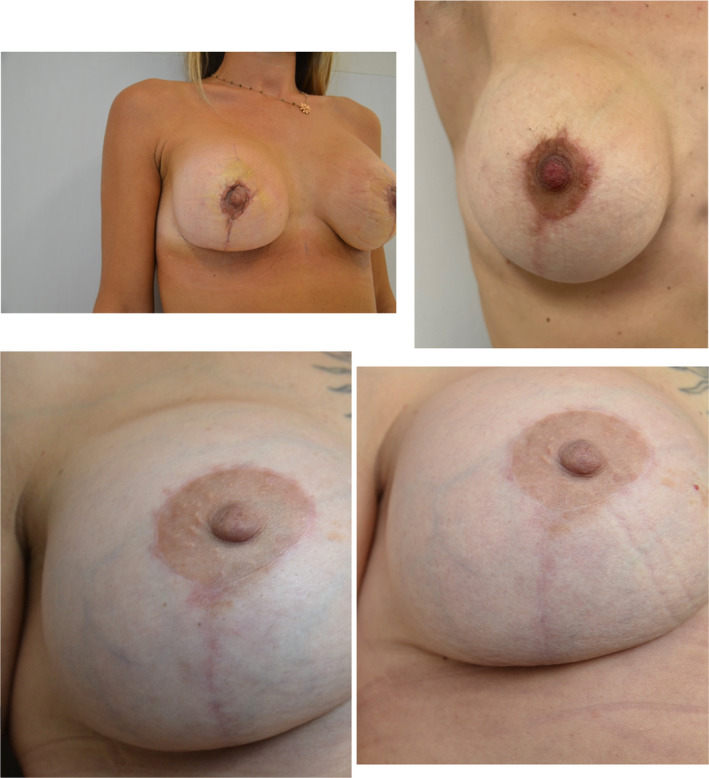
Aesthetic improvement of two postsurgical scars (from above, patients 13 and 19) in the mammary areolar area at baseline (left) and at the end of the PN HPT treatment (right)—courtesy of Dr. Antonino Araco with the preliminary written consent of treated subjects.

Figure 2 illustrates two examples of the macroscopic appearance of postsurgical mammary scars before and after the five intradermal PN HPT treatment sessions in two cohort women treated between September 2022 and February 2023.

### 3.2 Secondary Retrospective Efficacy Parameters

**FIGURE 3:**
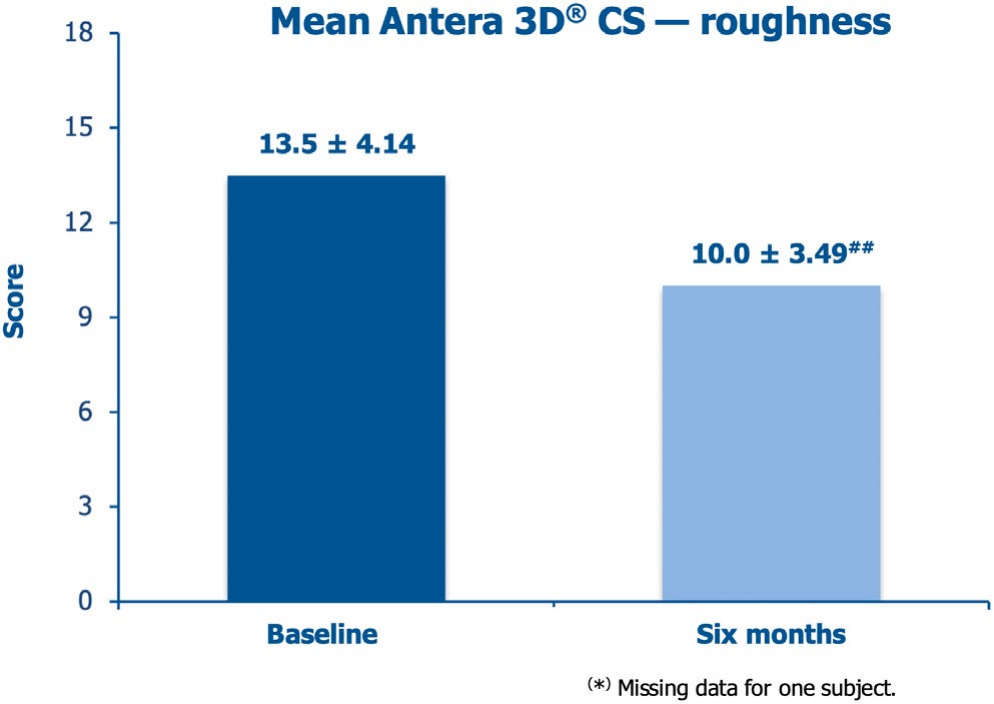
Mean Antera 3D CS roughness scores at baseline and the final sixth‐month visit (^#^ *p* < 0.01 vs. baseline).

The severe mean Antera 3D CS roughness subscore at baseline, 13.5 ± 4.14, improved by a clinically appreciable 25.9% after 6 months of follow‐up (Figure 3). The Antera 3D CS pliability subscore, falling from 3.1 ± 0.43 at baseline to 1.8 ± 0.83 and 1.9 ± 0.80 after three and 6 months, highlighted the seemingly rapid, objective improvement of the scar evidence, with smoother appearance and better blending with the surrounding skin.

**FIGURE 4:**
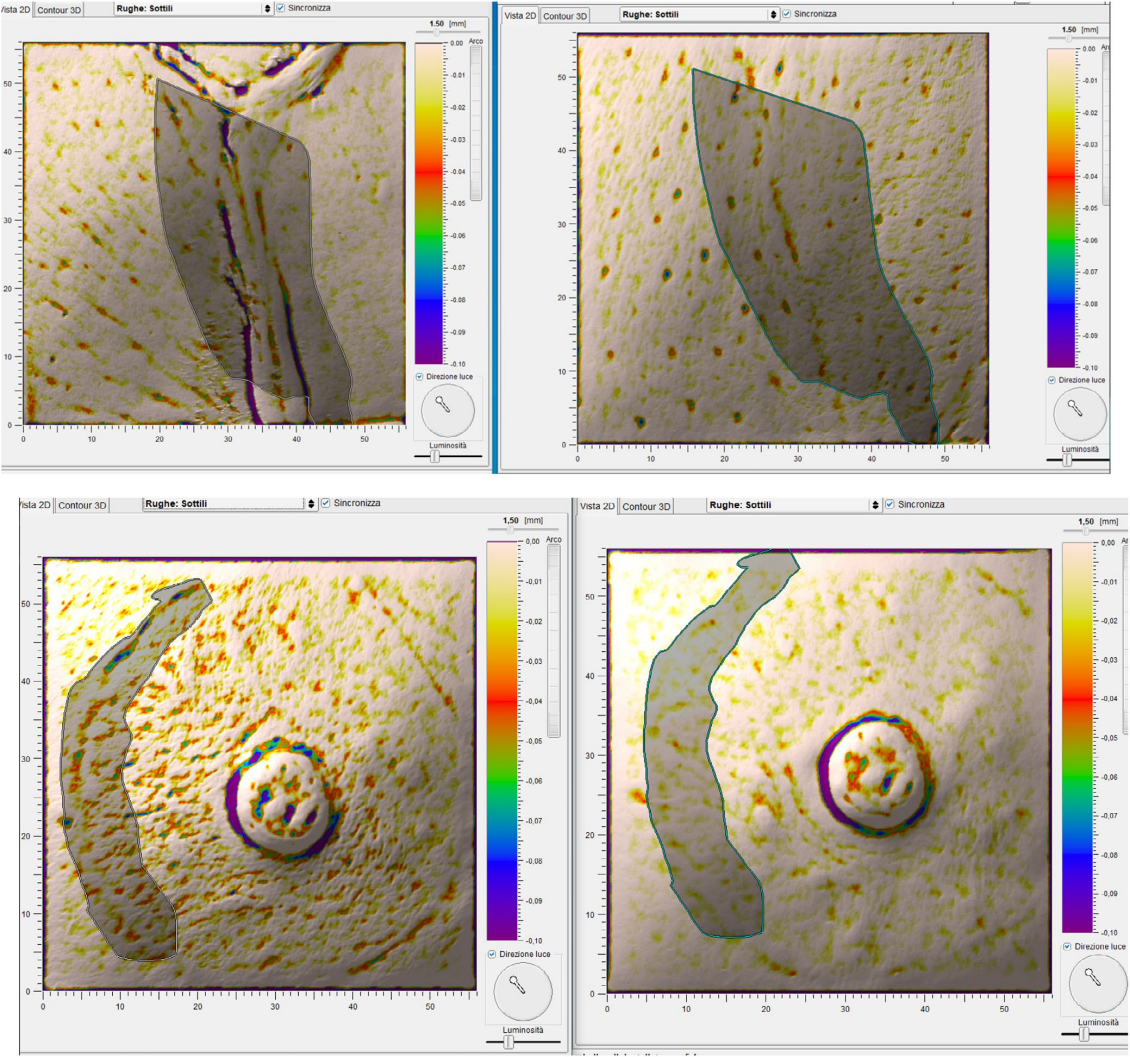
Improvement of the postsurgical scar roughness (from above, patients 1 and 11) as measured by the notching index at baseline (left) and at the end of the PN HPT treatment (right)—courtesy of Dr. Antonino Araco with the preliminary written consent of treated subjects.

**FIGURE 5:**
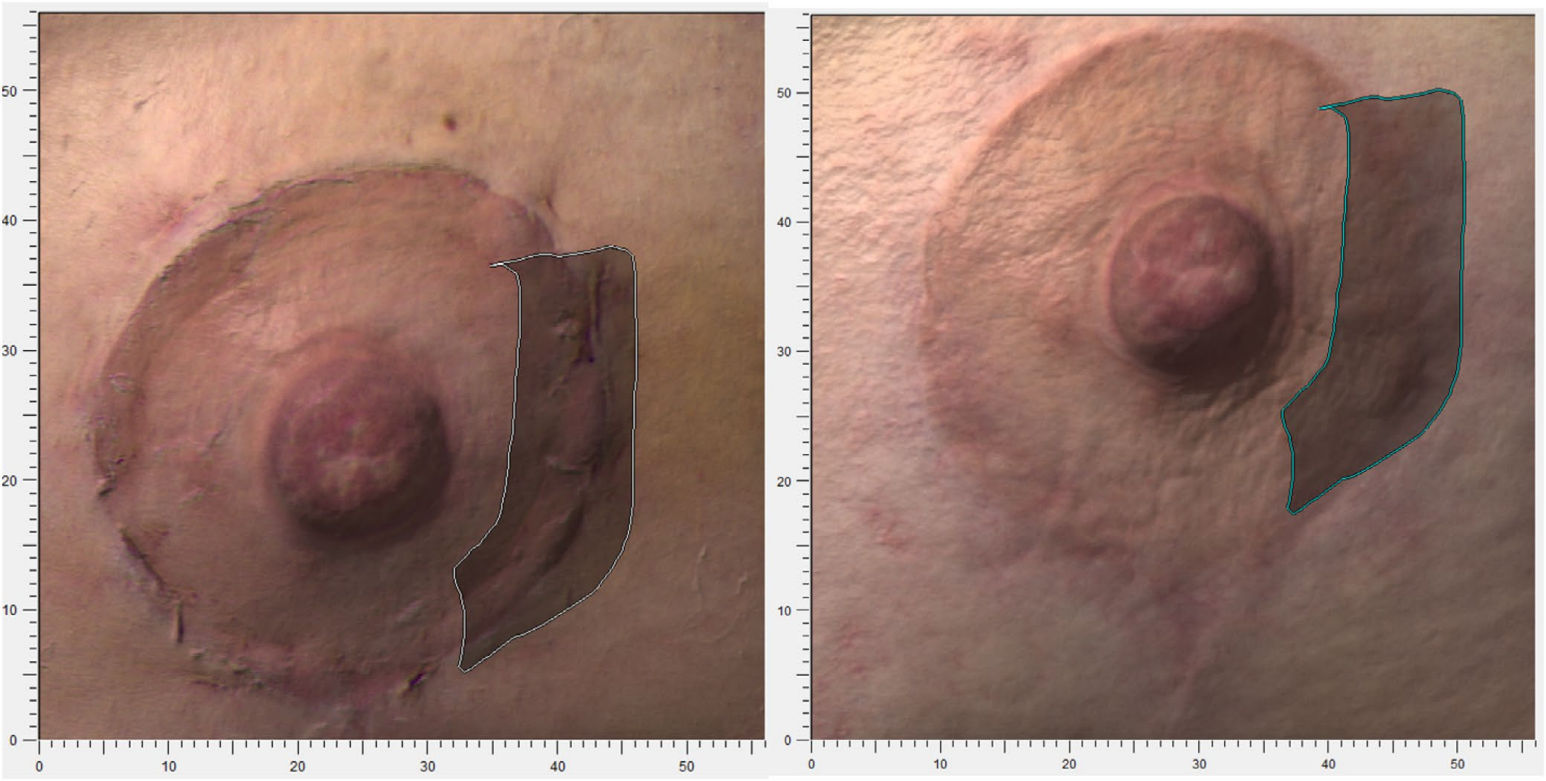
Reduced pigmentation of the postsurgical mammary scar compared with the surrounding normal skin (patient 3: Baseline on the left, six‐month assessment on the right spectral channel; Antera 3D CS system)—courtesy of Dr. Antonino Araco with the preliminary written consent of treated subjects.

Figures 4 and 5 show the improvements, objectively assessed with Antera 3D CS, in the roughness and pigmentation of the postsurgical scars in three representative cohort women at the final 6‐month follow‐up visit. The roughness scores improved from 10.9 and 12.2 to 5.83 and 5.40 in the upper and lower tridimensional images; the less sharp scar pigmentation allowed a better blending with the surrounding skin.

### 3.3 GAIS Assessment (Investigator and Patient Subscales)

After three and 6 months, the GAIS subscores were identical for investigators and cohort subjects (3.0 ± 0.81 and 3.0 ± 0.72, respectively). Table 6 analytically shows the lack of differences in the proportions of GAIS responders (subjects improving by at least one grade at the final follow‐up visit compared to baseline) according to investigators and treated subjects, with most subjects improving objectively and subjectively after 6 months: 50% by one GAIS grade (“Improved”) and 27% by two GAIS grades (“Much improved”).

**TABLE 6:** GAIS evaluations by the investigators and the thirty retrospective cohort subjects, final six‐month follow‐up visit.

GAIS descriptor	Investigator		Patient	
Worse	0	0%	0	0%
No change	7	23%	7	23%
Improved	15	50%	15	50%
Much improved	8	27%	8	27%
Optimal results	0	0%	0	0%

### 3.4 Safety

There were only some occasional reports of mild and transient swelling and pain, expected after an intradermal injection and reported in the product information leaflet, with no severe or unpredictable adverse event.

## 4 Discussion

Scars are often a significant concern for postsurgical patients, with ideal scars essentially undetectable and similar in texture, roughness, and color to adjacent tissues. With meticulous operative techniques, targeting one or more of the three phases of wound healing, inflammation, proliferation, and remodeling to create an ideal environment for wound healing is paramount to lessening the aesthetic and symptom burden of postoperative scars [19].

With a clearly stated, purely exploratory ambition, the study aimed to retrospectively verify the efficacy of a five‐session intradermal PN HPT treatment course over 6 months. It enrolled 30 women, mainly in their forties, seeking an objectively and subjectively meaningful aesthetic relief of their postsurgical mammary scars. Despite the lack of a retrospective control group and the open‐label design, admittedly both weak points of the study, the consistently favorable outcomes appear worthy of at least an indicative trust thanks to the relatively high number of postsurgical mammary scars and the use of the modified Vancouver Scar Scale and GAIS, two widely used validated scoring tools. Although the outcomes, especially the magnitude of the benefits, cannot be overestimated since the retrospective nature and the lack of a formal parallel control group—not even a split‐face design—remain severe biases, assessing the scar roughness quantitatively by Antera 3D CS advanced digital technology allowed the investigators to counterbalance the severe design limitations, at least partially. The 6‐month follow‐up period, which allowed discrimination between short‐term and evanescent effects and long‐term modulation of the scar structure, is another commendable point of the study.

Regarding mVSS, the literature considers clinically significant a reduction of at least 20% of the total mVSS score, the primary retrospective efficacy parameter [10, 11]. The mean improvements of about 38% after 3 months and 39% after 6 months compared favorably with the mVSS threshold, although always with the limitations of a missing control group and the intrinsic bias due to a semiquantitative subjective scoring scale. The objective Antera 3D CS scar measurements, which demonstrated roughness improvements in all patients, are more trustworthy and solid than possibly biased, subjective semiquantitative scores, with a mean roughness score reduction of more than one‐fourth at the final 6‐month follow‐up visit.

The endogenous tissue DNAses release the nucleotides and other nucleic acid precursors known to be physiologically present in the cell interstitium [8]. The indirect, non‐pharmacological fibroblast activation by PN HPT, with deposition of type‐III and then persistently type‐I collagen, counteracts the collagen destruction following reactive inflammation occurring in atrophic and depressed postsurgical wounds and is the rationale for the long‐term PN HPT efficacy. Independently of the clinical presentation, the same applies to atrophic acne scars because the inflammatory events leading to collagen destruction are similar [6, 7, 16].

New collagen deposition is a feature that the PN HPT ingredient shares with other energy‐based and non‐energy‐based treatment options for depressed atrophic scars. Still, the rapid onset of the aesthetic benefits may be distinctive. Three months of follow‐up and a month after the end of the treatment cycle was enough for the total mean mVSS to improve by more than a third from baseline. With due caution, the GAIS subscore analysis seems to reveal that pliability (−33.3%) and scar height (−41.9%) were the two morphological determinants that most improved at short‐term follow‐up, together with almost complete symptom relief (scar pain −90%, itchiness −97%). The immediate hydrating and tissue‐expanding effect probably also had a role.

The study also confirmed that PN HPT intradermal injections are usually pain‐free, with minor edema and bruising as the only occasional side effects [6]. In the future, prospective and adequately designed confirmatory studies will overcome the liabilities of this retrospective exploratory study.

## Author Contributions

Dr. Antonino Araco and Dr. Francesco Araco performed all office procedures and contributed to the retrospective study design and medical writing; Dr. Mauro Raichi contributed to the retrospective study design, statistical analyses, and medical writing. All authors agreed to the manuscript submitted to the Journal of Cosmetic Dermatology and are accountable for its clinical and editorial accuracy and integrity. The authors confirm that they adhered to the journal's ethical policies.

## Disclosure

Over the past 36 months, Dr. Antonino Araco and Dr. Francesco Araco received research grants and consultancy fees from Mastelli S.r.l. and other producers of fillers and aesthetic medicine/plastic surgery products as lecturers at Continuous Medical Education activities and other sponsored educational meetings or producers of educational materials. Dr. Mauro Raichi received consultancy fees from Mastelli S.r.l. and international communications networks for medical education activities and educational materials. The authors confirm no other conflicts of interest related to their retrospective study.

## Ethics Statement

All activities reviewed in the manuscript were within the regulatorily accepted indications in the patient Information leaflets of administered devices. According to accepted regulatory requirements (i.e., Article 62 to Article 82 of the European Union regulation MDR 2017/745 and Article 16 of the Legislative Decree 137/2022), these considerations would have allowed waiving the requirement for formal approval by an Ethical Committee when absent—even more so for Polynucleotides HPT‐based medical devices still regulatorily classified in the MDD category (The Medical Device Directive—Council Directive 93/42/EEC of 14 June 1993).

## Consent

All photographs belong to the contributing author named in the caption and were taken by him during his office‐based treatments. The contributing author agrees to their submission and publication. The treated subjects agreed to publish their photographs when they signed their preliminary informed consents, provided all subjects were unrecognizable.

## Conflicts of Interest

The authors declare no conflicts of interest.

## Data Availability

The data that support the findings of this study are available on request from the corresponding author. The data are not publicly available due to privacy or ethical restrictions.
